# More Than a Feeling—Interrelation of Trust Layers in Human-Robot Interaction and the Role of User Dispositions and State Anxiety

**DOI:** 10.3389/fpsyg.2021.592711

**Published:** 2021-04-12

**Authors:** Linda Miller, Johannes Kraus, Franziska Babel, Martin Baumann

**Affiliations:** Department Human Factors, Institute of Psychology and Education, Ulm University, Ulm, Germany

**Keywords:** trust in robots, human-robot interaction, trust layers, user dispositions, affect, state anxiety, comfort distance, trust in automation

## Abstract

With service robots becoming more ubiquitous in social life, interaction design needs to adapt to novice users and the associated uncertainty in the first encounter with this technology in new emerging environments. Trust in robots is an essential psychological prerequisite to achieve safe and convenient cooperation between users and robots. This research focuses on psychological processes in which user dispositions and states affect trust in robots, which in turn is expected to impact the behavior and reactions in the interaction with robotic systems. In a laboratory experiment, the influence of propensity to trust in automation and negative attitudes toward robots on state anxiety, trust, and comfort distance toward a robot were explored. Participants were approached by a humanoid domestic robot two times and indicated their comfort distance and trust. The results favor the differentiation and interdependence of dispositional, initial, and dynamic learned trust layers. A mediation from the propensity to trust to initial learned trust by state anxiety provides an insight into the psychological processes through which personality traits might affect interindividual outcomes in human-robot interaction (HRI). The findings underline the meaningfulness of user characteristics as predictors for the initial approach to robots and the importance of considering users’ individual learning history regarding technology and robots in particular.

## Introduction

Once utopian, robots are increasingly finding their way into public and private settings to assist humans in everyday tasks. Thereby, service robots offer numerous potentials for improvements in many fields, for example, by supporting disabled people to live more independently (e.g., Robinson et al., 2014). In these upcoming environments, robots represent a rather new and unfamiliar technology that most people have no specific knowledge or personal experience with. As many of these application areas for robots are characterized by increased complexity, dynamic, and interaction with untrained novice users, the interaction design needs to account for more flexibility and adaptability to both changing surroundings and users. Regarding the adaptability to users, it is a specifically important endeavor to reduce uncertainties and negative psychological consequences to facilitate an appropriate and repeated interaction with robots.

Based on interaction norms between humans, people treat robots as social partners in many respects. Thus robots are expected to behave in a socially acceptable manner and comply with social rules to some extent (e.g., Computers Are Social Actors paradigm, Nass et al., 2000; Nass and Moon, 2000; Rosenthal-von der Pütten et al., 2014). Thereby, amongst others, user characteristics (e.g., personality, Walters et al., 2005) were found to influence the individual reaction to robots. For example, such individual differences for the preferred proximity are discussed in Leichtmann and Nitsch (2020). At this point in human-robot interaction (HRI) design, psychological mechanisms need consideration to achieve positive interaction outcomes.

A multitude of research emphasized the importance of *trust* in the initial encounter with automated technologies (Lee and See, 2004; Stokes et al., 2010; Hoff and Bashir, 2015). Building on that, this research examines the role of this psychological variable that has also been thoroughly discussed and investigated in regard to the interaction with robots (e.g., Hancock et al., 2011; Schaefer, 2013; Salem et al., 2015), conceived in this context as advanced, complex automated technical systems. The research field of trust in automated systems is, amongst others, rooted in the observation that people do not use automation appropriately (Lee and See, 2004). Inappropriate use can be reflected in too much trust (overtrust), leading to misuse of a system on the one hand and too little trust (distrust), leading to disuse of a system on the other hand (Parasuraman and Riley, 1997; Lee and See, 2004). Thereby, to achieve an optimal, efficient, and safe interaction instead of an unconditional maximization of trust, designers might aim to achieve a calibrated level, which corresponds to a system’s actual capabilities (calibrated trust; Muir, 1987). A calibrated level of trust has been related to a balanced usage of and reliance to innovative autonomous technology, thus facilitating a successful long-term relationship.

A good deal of research on trust in automation has focused on aviation and automated driving systems. While many of the findings in these and related areas might be readily transferred to HRI, this research seeks to validate and extend previous findings on the role of trust in automation to the interaction with robotic systems in domestic surroundings. Thereby, several specificities of domestic HRI have to be considered. First, domestic robots enter the user’s personal space—not only in the sense of operating in private homes but also in a spatial and proxemic way. Second, robots can move around more flexible and might manipulate objects. Third, the prototypical user is not a trained professional. Fourth, domain-specific individual preferences, attitudes, and emotions are discussed to play a role in processes for evaluating and adopting robots. Essentially, negative robot attitudes and fear of robots are commonly discussed as potential influencing factors for the adoption of robots (Nomura et al., 2008; Syrdal et al., 2009; Złotowski et al., 2017). Still, the relationship of these factors with trust in robots has, up to now, only scarcely been investigated. Taken together, these particularities of HRI have to be kept in mind when comparing and transferring findings from other domains to the interaction with (domestic) robots.

In the presented study, the role of user dispositions and individual experiences of anxiety in the face of an unfamiliar robot on trust formation and proximity preferences has been investigated. This research focus is based on the overall assumption that general user dispositions affect the experiences throughout an individual’s learning history with technology. This, on the one hand, leads to the formation of more specific technology-related personality traits and attitudes. On the other hand, the manifestation of an individual’s dispositions in system-specific attitudes and behavior is expected to be subject to fluctuations and shaped by the affective state in a situation. In the presented laboratory experiment, users encountered a domestic service robot and indicated their state anxiety, trust, and comfort distance toward the robot. Based on recent findings on the role of individual levels of anxiety in the familiarization process with unfamiliar automated driving systems (e.g., Kraus et al., 2020a), this research is the first of its kind to extend the investigation of such a relationship to the domain of service robots. On this basis, suggestions for the design of initial interactions with robots in domestic environments are derived.

## Theoretical Background

### Trust in Automation and Robots

Like in interpersonal relationships, trust is a fundamental requirement for successful human-machine interaction guiding decisions in unknown and risky situations (e.g., Lee and See, 2004; Hoff and Bashir, 2015). This is reflected in the definition of trust in automation as “the attitude that an agent will help achieve an individual’s goal in a situation characterized by uncertainty and vulnerability” (Lee and See, 2004, p. 51). On a conceptual level, trust is assumed to influence the behavior in regard to automation as part of a dynamic feedback loop, while the automation’s attributes and actions also affect the level of trust (Lee and See, 2004). To establish an enhanced understanding of the complex psychological processes in which trust is formed, calibrated, and related to reliance decisions, a differentiation between several trust concepts seems worthwhile.

In their review on trust in automation, Hoff and Bashir (2015) conceptually distinguish different trust layers. Based on Kraus’ (2020) *Three Stages of Trust* framework, an integration and extension of Lee and See’s (2004) and Hoff and Bashir’s (2015) models, three trust layers can be distinguished: the propensity to trust in automation, initial learned trust, and dynamic learned trust. First, the propensity to trust in automation (dispositional trust in Hoff and Bashir, 2015) refers to an automation-specific form of dispositional trust. The latter was defined in the interpersonal domain as “a diffuse expectation of others’ trustworthiness […] based on early trust-related experiences” (Merritt and Ilgen, 2008, p. 195). Building on this, the propensity to trust refers to a general context- and situation-independent personal predisposition to trust in automated technology (e.g., Hoff and Bashir, 2015). In the Three Stages of Trust framework, it is proposed that this trust layer is established from the combined influences of users’ dispositions (e.g., demographics, culture, personality, and general technology attitudes) and the individual learning history with technology. Accordingly, users with a comparatively higher level of the propensity to trust in automation are more likely to be more trusting in the evaluation and interaction with unfamiliar automated systems, for example, robots (Kraus, 2020).

In contrast to the propensity to trust, *learned trust* comprises trust in a specific system. In learned trust, available information about a given system’s trustworthiness is used to assess the system’s trustworthiness in a situation of uncertainty (Kraus, 2020). This trustworthiness expectation is considerably informed by available diagnostic information—so-called trust cues—which were described as “in some way observable or given pieces of evidence a trustor might use to draw inferences about a trustee’s trustworthiness in a specific situation” (Thielmann and Hilbig, 2015, p. 21). Kraus (2020)—based on Lee and Moray (1992) and Thielmann and Hilbig (2015)—proposes that the available information during trust formation can be differentiated into five categories along with the included trust cues: reputation-, purpose-, process-, performance-, and appearance-related. In the trust calibration process, the acquisition of new information affects the level of learned trust to the extent to which it derivates from the current trustworthiness expectation.

Within learned trust, one can further distinguish between *initial learned trust* based on information and existing knowledge prior to the interaction with a system and *dynamic learned trust*, which refers to trust adaptions during the actual interaction with a given system. It follows that learned trust is subject to change over time and is updated before and during the interaction by accumulated information and observations, for example, on perceived system performance (Merritt and Ilgen, 2008; Kraus et al., 2019b). It is further assumed that this process of learning to trust follows to a considerable extent the mechanisms of attitude formation and change (see Maio et al., 2018, exemplarily).

In the process of trust formation and calibration, the influences of many variables have been investigated in different technological domains. This is nicely summarized in several meta-analyses and reviews (see, e.g., Lee and See, 2004; Hancock et al., 2011; Hoff and Bashir, 2015; Schaefer et al., 2016). The different variables fall into the categories: person-related (e.g., personality, expertise, demographics), system-related (e.g., reliability, functionality, design), and situation-related (e.g., workload, affect). In the early days of research on trust in automation, the dynamic trust development in process control simulation micro-worlds was a central focus (e.g., Lee and Moray, 1992, 1994; Muir and Moray, 1996). More recently, trust processes were also investigated in the domains of automated driving (e.g., Hergeth et al., 2016), information technology (e.g., McKnight et al., 2002), and robots (e.g., Hancock et al., 2011).

This research’s central focus is investigating the interrelation between the propensity to trust, initial learned trust before the interaction with a robot, and dynamic learned trust, developing and dynamically adapting during the interaction with a robot. The two forms of learned trust are thereby assumed to emerge in an attitude-formation process and then be calibrated along with a comparison of expectations with a robot’s behavior. A detailed theoretical discussion of the psychological processes of formation and calibration of trust in automation is provided in the Three Stages of Trust framework (Kraus et al., 2019b; Kraus, 2020). A central assumption of this framework is that interindividual differences in trust are to some degree based on personality differences and the technology-related learning history of users, which affect the feelings toward and evaluation of a specific technological system (e.g., a robot).

### Investigated Relationships

**FIGURE 1:**
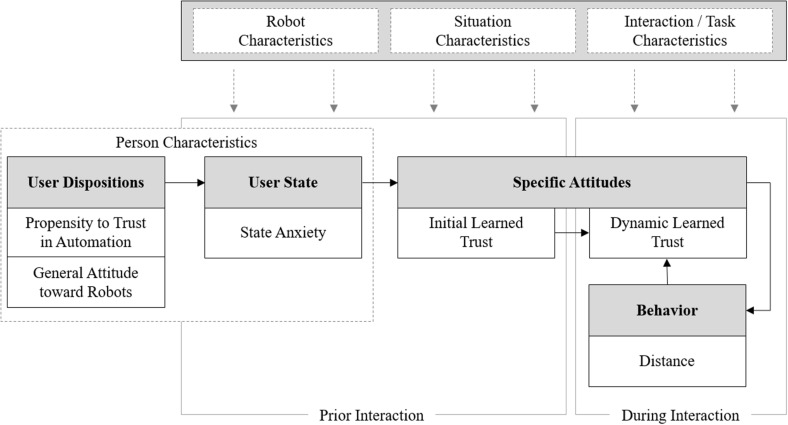
Study framework based on the Three Stages of Trust (Kraus, 2020).

Following Kraus’s (2020) framework, this research takes an integrative approach in the investigation of the interrelation of different trust layers (Figure 1). It is proposed that person characteristics (e.g., user dispositions and states) influence learned trust, which in turn builds a basis for behavior in HRI. In line with other trust models (e.g., Lee and See, 2004), the investigated model is rooted in the *Theory of Planned Behavior* (Fishbein and Ajzen, 1975; Ajzen, 1991), which assumes that behavior is determined by a cascade of beliefs, attitudes, and intentions. It is expected that characteristics of the situation (e.g., physical and legal attributes), the robot (e.g., appearance, performance, communication capabilities), and the task and interaction itself (e.g., goal, type) will moderate this process. Based on this, the hypotheses of this research are derived from theory and empirical findings in more detail below.

### Trust as a Function of User Dispositions and States

This research builds on the general differentiation between cross-situational *traits*, which are comparatively stable person characteristics (e.g., personality), and short-term *states* (e.g., affect). States are situation-specific and reflect a person’s adaption to given circumstances (e.g., social and physical situation, physiological and cognitive processes; Hamaker et al., 2007). Building on the assumption that traits and states contribute to interindividual variation in trust and behavior (e.g., Buss, 1989; Kraus, 2020), this research focuses on user characteristics as antecedents of trust. In line with this, trust in automation was found to be essentially influenced by both user traits and states (Merritt and Ilgen, 2008; Kraus et al., 2020a, b).

Above this, the inclusion of affect as a potential antecedent of trust in automation can be established on the basis of the *affect-as-information* model (e.g., Schwarz and Clore, 1988). This model proposes that people use their current affective state as an information basis for judgments about an object under consideration. Accordingly, various research has shown the impact of emotional and affective states on attention, perception, judgments, attitudinal responses, and behaviors in human interaction (e.g., Forgas and George, 2001; Brief and Weiss, 2002; Dunn and Schweitzer, 2005; Forgas and East, 2008). In conclusion, robot-related psychological outcomes such as trust are expected to be attenuated by users’ affective states in a state-congruent direction (Brave and Nass, 2007). Taken together, it is proposed that users’ state anxiety before and during an interaction affects their learned trust in a robot. This mechanism is expected to differ between users based on their dispositional propensity to trust in automation and attitude toward robots in general.

#### Traits, Attitudes, and Trust

Research on the influence of user characteristics on trust in HRI is relatively scarce (Hancock et al., 2011; Lewis et al., 2018). The inconclusive results on the relationship between personality traits and trust in automation call for further studies with well-founded theorizing and methodological quality to gain insight into the actual influence of personality factors in HRI (see, e.g., Kraus et al., 2020a, for a discussion of the foundation of trust in automation in personality traits). For this, a conceptual distinction between dispositional personality traits and attitudes provides an essential starting point for hypothesizing. According to Ajzen (2005), the trait and attitude concepts share substantial similarities (e.g., manifestation in observable responses) and differ regarding stability and focus. Following the definition of Ajzen (1989), *attitudes* can be understood as “an individual’s disposition to respond favorably or unfavorably to an object, person, institution, or event” (p. 241). Whereas attitudes entail an evaluation of an object which is more prone to be changed, for example, by new information, personality *traits* refer to “response tendencies in a given domain” (Ajzen, 2005, p. 6) and are not oriented toward a specific object. Both traits and attitudes can differ in their specificity, as reflected in domain-specific traits (e.g., the propensity to trust in automation) and attitudes (e.g., attitude toward robots). A combined consideration of both technology-related traits and attitudes is valuable for explaining individual differences in HRI. Based on this reasoning, in this research, the domain-specific personality trait propensity to trust in automation is integrated along with the global attitude toward robots on the dispositional level, predicting user state, learned trust, and proxemic preferences.

#### Propensity to Trust in Automation

The dispositional layer of trust in automation, the *propensity to trust in automation*, can be defined as “an individual’s overall tendency to trust automation, independent of context or a specific system” (Hoff and Bashir, 2015, p. 413). Following Mayer et al. (1995), the propensity to trust automation is hypothesized to influence learned trust. This relationship was supported before by empirical findings on a positive association between the dispositional propensity to trust and system-specific initial and dynamic learned trust (e.g., Merritt and Ilgen, 2008; Merritt et al., 2013; Kraus et al., 2020a). In line with this, in the domain of HRI, Tussyadiah et al. (2020) recently reported that users with a higher propensity to trust in technology in general hold more trusting beliefs toward a service robot. In addition to these findings, the presented research investigates the relationship between the three trust layers propensity to trust in automation, initial, and dynamic learned trust in a service robot. Accordingly, it was hypothesized that:

Hypothesis 1: The propensity to trust in automation is positively related to initial and dynamic learned trust in a robot (H1.1). The effect of propensity to trust on dynamic learned trust is mediated by initial learned trust (H1.2).

#### Negative Attitude Toward Robots

Besides the propensity to trust, the predictive power of domain-specific attitudes for trust formation was supported in previous research. In automated driving, the prior attitude toward automated driving systems was linked to trust in automation in several studies (Singh et al., 1993; Merritt et al., 2013; Kraus et al., 2020a). Also, in the robotic domain, the role of (negative) *attitudes toward robots* was investigated before (e.g., Nomura et al., 2006a, b; Syrdal et al., 2009; Tsui et al., 2010). Thereby, previous research underlined the role of robot attitudes for the evaluation and interaction with robots (e.g., Nomura et al., 2007; Cramer et al., 2009; Syrdal et al., 2009; De Graaf and Allouch, 2013b). Yet, studies on the relationship between attitudes toward robots and trust layers arrived at inconclusive results. While Sanders et al. (2016) could not find an association between implicit attitudes and dynamic learned trust, Wang et al. (2010) reported lower dynamic learned trust to be associated with a more negative attitude toward robots. In line with the latter, Tussyadiah et al. (2020) reported a strong negative association between a general negative attitude and trusting beliefs in a service robot. While these mixed findings might be in part resulting from conceptual underspecifications of both the included attitude and trust variables, in this research in line with the proposed conceptual differentiation of trust layers and between traits and attitudes, a relationship between prior domain attitudes and learned trust was hypothesized:

Hypothesis 2: Negative attitudes toward robots are negatively related to initial and dynamic learned trust in a robot (H2.1). The effect of negative attitudes toward robots on dynamic learned trust is mediated by initial learned trust (H2.2).

Furthermore, the degree of experience and familiarity with an interaction partner is expected to influence users’ trust levels. The more often one interacts with a partner, the better and more realistically the trustworthiness can be evaluated and aligned with one’s own experiences. This is supported by numerous findings regarding trust in automation in general and trust in HRI in particular, which show trust to increase over time with repeated error-free interaction and growing familiarity (e.g., Muir and Moray, 1996; Beggiato and Krems, 2013; van Maris et al., 2017; Yu et al., 2017; Kraus et al., 2019b). Therefore, it is hypothesized that:

Hypothesis 3: Learned trust in a robot increases with repeated error-free interaction.

#### The Role of State Anxiety for Trust in Robots

Besides user dispositions, users’ emotional states during the familiarization with a robot are a potential source of variance for robot trust. As the experience of emotional states has been shown to be considerably affected by personal dispositions, this research proposes a general mediation mechanism from the effects of user dispositions on trust in automation by user states (see Figure 1).

The presented research focuses on *state anxiety* as a specific affective state, which is expected to explain interindividual differences in trust in robots (e.g., Nomura et al., 2007; Kraus et al., 2020b). State anxiety is defined as “subjective, consciously perceived feelings of apprehension and tension, accompanied by or associated with activation or arousal of the autonomic nervous system” (Spielberger, 1966, p. 17). It is posited to “initiate a behavior sequence designed to avoid the anger situation or […] evoke defensive maneuvers which alter the cognitive appraisal of the situation” (Spielberger, 1966, p. 17). Thereby, state anxiety was found to selectively direct attention to anxiety-triggering stimuli (Mathews and MacLeod, 1985; MacLeod and Mathews, 1988). Following the reasoning of the affect-as-information approach, users who encounter a robot for the first time might use their emotional states to build their trust toward the unfamiliar technology.

Regarding trust in interpersonal relationships, emotional states were found to influence a person’s trust level (Jones and George, 1998; Dunn and Schweitzer, 2005; Forgas and East, 2008). For example, the results from Dunn and Schweitzer (2005) indicate that positive emotional states (e.g., happiness) positively and negative emotional states (e.g., anger) negatively affect trust in an unfamiliar trustee. Moreover, affective states were found to be related to trust in different automated systems (e.g., state anxiety, Kraus et al., 2020b; positive and negative affect, Stokes et al., 2010; Merritt, 2011). Interestingly, Stokes et al. (2010) found that affect was especially relevant in early trust formation processes. The relative influence diminished after repeated interaction and was replaced by more performance-related cues. These findings are in line with Lee and See’s (2004) assumptions, who claimed initial trust levels to follow affective processing, and subsequent trust to be guided more by analytical processes (including, e.g., perceptions of system performance). Accordingly, the results of Kraus et al. (2020b) indicate that state anxiety predicts trust differences. Thereby, anxiety was a stronger predictor for trust in automation than negative and positive affect. As throughout the interaction more specific and tangible information about the robot becomes available, it is further hypothesized that the effects of emotional states on the actual level of learned trust diminish. Taken together, it was hypothesized:

Hypothesis 4: State anxiety is negatively related to initial learned trust in a robot (H4.1). The relationship between state anxiety and learned trust in a robot diminishes with repeated interaction (H4.2).

In the interpersonal context, people with low dispositional trust are assumed to expect others to be dishonest and potentially dangerous (Gurtman, 1992; Mooradian et al., 2006). As anxiety might facilitate oversensitivity and overinterpretation of potential threats and risk, it might mediate the link of the propensity to trust and learned trust. This assumption is supported by the findings of Kraus et al. (2020b), which show a mediation effect of state anxiety between several personality traits and dynamic learned trust in the interaction with an automated driving system. Taken together, it was expected that:

Hypothesis 5.1: The relationship between the propensity to trust in automation and initial learned trust in a robot is mediated by state anxiety (H5.1).

Above this, it is assumed that attitudes toward robots reflect the overall evaluation emerging, for example, from the assessment of their utilitarian and hedonistic benefits (Brown and Venkatesh, 2005; De Graaf and Allouch, 2013a). Several studies reported a high positive correlation between negative attitudes toward robots and state anxiety (e.g., Nomura et al., 2006b, 2008). Based on this, a mediation of the effect of negative robot attitudes on trust in a specific robot by state anxiety was hypothesized as follows:

Hypothesis 5.2: The relationship between negative attitudes toward robots and initial learned trust in a robot is mediated by state anxiety (H5.2).

### Trust and Distancing Behavior

In this study, interindividual comfort zones toward robots were investigated, whereas distancing behavior was adopted as an objective interaction behavior. Spatial proximity is an essential part of human relationships. People prefer to maintain a personal space around themselves, which is expected not to be violated by others (see Hayduk, 1978, for an overview). A violation of the personal space may lead to the experience of threat and discomfort (Hayduk, 1978; Perry et al., 2013). Therefore, robot proxemic behavior design is vital for establishing close relationships and comfortable collaborations between humans and robots.

To explain distancing behavior and its function, different approaches of human relations can be drawn on (see Leichtmann and Nitsch, 2020, for an overview). Following the affect-as-information approach, an *arousal-regulating function* can be ascribed to interpersonal distancing behavior to prevent an information overload and maintain a balanced arousal level (Aiello, 1987; Leichtmann and Nitsch, 2020). Hence, arousal models argue for the change of arousal level due to interaction and approach, which leads to a cognitive evaluation and behavioral adaption (Aiello, 1987). In line with this, personal spaces can be seen as a function of perceived threat, with external sources of threat causing larger distances (anxiety-defense process, Meisels and Dosey, 1971). Regarding the interaction with robots, distancing behavior’s arousal-regulatory function could play a significant role in the first encounter with this unfamiliar sophisticated technology to reduce unpleasant affective states. In this research, it is assumed that people will adapt their comfort zone toward the robot based on their initial trust level.

As theorized in different models on trust in automation (Lee and See, 2004; Hoff and Bashir, 2015; Kraus, 2020), trust has been found to be a major antecedent of reliance and, thus, of behavioral outcomes in the interaction with various technological systems (Lee and Moray, 1994; Muir and Moray, 1996; Lewandowsky et al., 2000; Hergeth et al., 2016; Payre et al., 2016). In line with this, the study by Babel et al. (2021) found a strong negative correlation between spatial distance and initial trust in a robot in a human-robot approach paradigm. The underlying mechanism of this association might be a feedback process (as proposed in the investigated research model, Figure 1), in which the repeated interaction with a robot might lead to an adjustment of trust based on previous interaction outcomes. In this regard, the experience made in a certain distance from the robot might influence trust, which in turn informs subsequent proximity decisions. Such a feedback process was supported by findings from MacArthur et al. (2017). In the same manner, Kraus et al. (2019b) reported a dynamic adaption of trust over the course of interaction to changing circumstances such as system malfunction. Deduced from this, it was hypothesized that:

Hypothesis 6: Initial learned trust and dynamic learned trust in a robot are negatively related to the comfort distance toward a robot.

Similar to trust, experience and familiarity with the interaction partner are assumed to influence proximity preferences positively. It is to be expected that people will interact in closer proximity with trusted than with untrusted partners (e.g., closer interaction with family and friends compared to strangers). In line with this, habituation effects were found by several authors concerning allowable distances, showing decreasing distances between people and robots with growing experience and familiarity (e.g., Koay et al., 2007; Haring et al., 2013; Lauckner et al., 2014). Therefore, it is hypothesized that:

Hypothesis 7: Comfort distance toward a robot decreases with repeated error-free interaction.

The proposed hypotheses were investigated in a laboratory study, in which lay users encountered and interacted with a domestic service robot for the first time in real life. In the following, the study methods, procedure, and design are depicted in more detail.

## Materials and Methods

In the presented laboratory experiment a trait-state-behavior mediation cascade in initial encounters with a domestic service robot was investigated to enhance the understanding of psychological mechanisms of trust formation and associated proximity preferences. Namely, the influences of user characteristics (dispositions and affective state anxiety) on learned trust and distancing behavior were analyzed in a repeated measure design. After the first familiarization with a humanoid robot, participants were approached by the robot two times and indicated their trust and comfort distance for each trial.

### Sample Characteristics

Participants were invited to meet a domestic robot for home assistance for the first time. They had to be fluent in German and be 18 years or above. In total, 34 participants took part in the study. After the exclusion of six participants (technical issues with the robot, three times; non-compliance with instruction, one time; univariate statistical outlier regarding distance, two times), the final sample for this study consisted of *N* = 28 participants (16 female) with an average age of *M* = 30.32 (*SD* = 13.61, ranging from 18 to 60 years). Participants’ technical affinity (scale of Karrer et al., 2009) was in an above-average range with *M* = 4.90 (*SD* = 1.21), trait anxiety (scale of Spielberger et al., 1970; Laux et al., 1981) on a rather medium level with *M* = 2.98 (*SD* = 0.81), both on a scale from 1 (totally disagree) to 7 (totally agree). Half of the sample indicated to be experienced with robots, which included industrial robots (*n* = 5) or vacuum cleaning robots (*n* = 10). Seven participants indicated owning a vacuum cleaning robot. None of the participants reported any personal experience or interactions with a humanoid service robot.

### Experimental Setup

**FIGURE 2:**
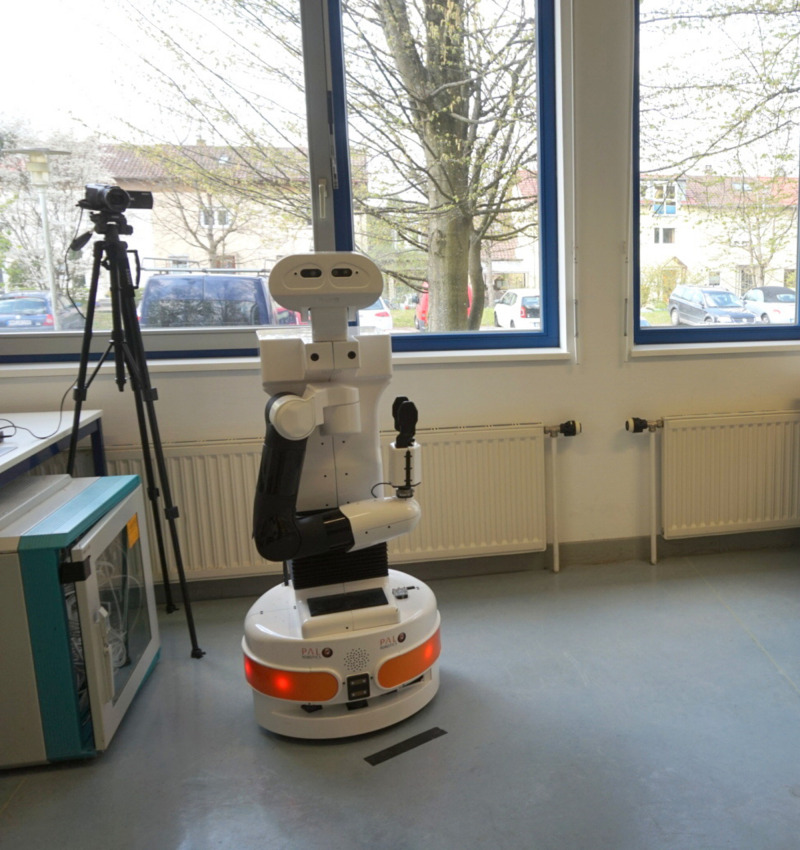
Investigated humanoid robot (TIAGo, PAL Robotics) in initial position with retracted manipulator.

While this study focuses on the correlative findings of the study, in the original design also an experimental manipulation was included, which is not part of this research but will now be described to ensure a complete picture of the study setup. In the laboratory experiment in a 2 × 2-mixed design, the manipulator outreach of a humanoid robot (TIAGo, see Figure 2) as a between-subject factor (retracted vs. extended; *n*_*retracted*_ = 14, *n*_*extracted*_ = 14) and the size of the robot as a within-subject factor (short vs. tall) were manipulated. In the extracted manipulator condition, the robot stretched out his arm at a right angle toward the participant, leading to a minimum distance between the participant and the robot of 0.63 m (measured from end of the mobile base to end-effector). In two trials, the robot drove toward the participant either in the short (1.10 m) or tall (1.45 m) height condition, which was randomized in order. Therefore, within the respective groups, half of the subjects faced the short robot first, the other half the tall robot. Considering the presentation order, four experimental counterbalanced groups resulted (*n* = 7 each). However, the results of the experimental manipulation are not part of this report and are described elsewhere (Miller et al., 2021).

### Procedure

**FIGURE 3:**
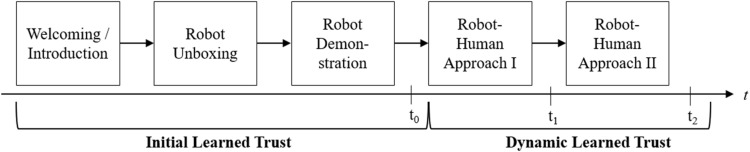
Study procedure of the laboratory study and times of measurement including the two layers of learned trust.

Figure 3 provides an overview of the overall study procedure. Participants were invited to meet a robotic housekeeping assistant for personal use. To account for the different trust layers and the timely sequence of the assumed relationships, initial learned trust was measured one time in the experimental scenario (t_0_), while dynamic learned trust and the distance preference were measured repeatedly (t_1_, t_2_). Figure 3 details how the different trust layers were successively addressed and measured in the study.

After providing informed consent, unboxing, and watching a short demonstration of the robot’s capabilities, participants took part in a semi-structured interview. After this, participants answered personality questionnaires and indicated their initial learned trust and state anxiety (t_0_). To check for the influence of subjective robot perceptions, different robot evaluations were assessed in advance as control variables. Table 1 provides an overview of the bilateral correlations of the robot evaluations, examined user charactersistics and dependent measurements. The experimental groups did not differ significantly in any of the listed measures.

**TABLE 1:** Descriptives of a priori robot evaluations and bivariate correlations with user dispositions, state anxiety and trust layers at the different times of measurement.

	*M*	*SD*	PTT	NARS	SA	ILT	DLT t_1_	DLT t_2_	DT t_1_	DT t_2_
Competence	4.55	1.16	0.36	0.17	**−0.47**	**0.41**	0.19	0.18	0.16	0.32
Anthropomorphism	3.24	1.14	0.24	−0.09	−0.07	0.37	0.16	0.15	0.23	0.15
Uncanniness	3.18	1.19	−0.33	**0.46**	**0.41**	**−0.63**	**−0.62**	**−0.62**	−0.16	−0.20

*Significant correlations in bold (p < 0.05, two-sided test). PTT, Propensity to Trust in Automation; NARS, Negative Attitude toward Robots; SA, State Anxiety; ILT, Initial Learned Trust; DLT, Dynamic Learned Trust; DT, Distance.*

For the part of the study, which is of interest here, the robot drove toward the participants (robot-human approach) two times. This was implemented with a Wizard of Oz paradigm, in which an operator remotely controlled and stopped the robot. In accordance with the stop-distance-technique (Hayduk, 1978) similarly applied in previous proximity studies in HRI (e.g., Koay et al., 2007; Syrdal et al., 2007), participants were instructed to stand on a marked spot in 3.70 m distance to the robot. They were asked to say “stop” as soon as they started to feel uncomfortable and wanted the robot to stop approaching (comfort distance; Leichtmann and Nitsch, 2020). After each trial, the experimenter measured the spatial distance and the participants indicated their dynamic learned trust (t_1_ and t_2_). The second trial immediately followed the first one. At the end of the study, participants answered questionnaires, including demographic variables and their previous experience with robots. In total, the study lasted around 45–60 min.

### Materials

#### Robot

For the study the humanoid robot TIAGo (PAL Robotics, see Figure 2) was used, which is variable in height (0.35 m torso lift, between 1.10 and 1.45 m) and has 12 degrees of freedom, e.g., it can move his head, arm (0.87 m reach), torso and mobile base. The robot was chosen to create consistency between the robot’s appearance and its application in a private domestic environment (e.g., Lohse et al., 2008). The maximum speed of the robot is 1 m/s, by which it approached the participants.

#### User Dispositions

The *propensity to trust in automation* was measured with four items of the Propensity to Trust Scale (Merritt et al., 2013). The adopted translation of Kraus et al. (2020a) shows high reliability (α = 0.91) and refers to “automated technology” instead of “machines” (e.g., “I usually trust automated technology until there is a reason not to.”). *Attitudes toward robots* were assessed with a self-translated eight-item version of the Negative Attitude toward Robots Scale (NARS) by Nomura et al. (2006c; e.g., “I would feel uneasy if robots really had emotions.”), which assesses humans’ overall attitude toward communicating robots. An English translation has shown acceptable psychometric quality (Tsui et al., 2010). Within this research, no differentiation between subscales was made, but an overall rating of the whole scale was used due to the ambiguous findings on the cultural fairness of the original loading structure (Syrdal et al., 2009; Tsui et al., 2010).

#### State Anxiety

The German short version of the State-Trait Inventory (STAI; Spielberger et al., 1970) translated by Laux et al. (1981) was used to assess the participants’ *state anxiety*. The state-scale (STAI-S) measures the cognitive and emotional components of anxiety as a state with five negatively and five positively poled items (e.g., “I feel tense.,” “I am calm.”). The STAI is considered a standard instrument in anxiety and stress research and shows high psychometric quality standards (Spielberger et al., 1970).

#### Learned Trust

*Initial learned trust* and *dynamic learned trust* were assessed as an unidimensional variable with the seven-item LETRAS-G (Kraus, 2020). Previous studies reported a high reliability of the scale (e.g., Kraus et al., 2019b). The items of the LETRAS-G were adapted to refer to “robots” instead of “automation” (e.g., “I trust the robot.”).

#### Distance

The robot’s spatial distance was measured in meters from the end of the robot’s mobile base to the subject’s toe after each trial. In the extended manipulator condition, 0.63 m (manipulator reach) were subtracted from the distance measure so that the value refers to the distance between the end-effector and the subject’s toe.

Except for the comfort distance, all constructs were assessed using self-report short-scales. All scales were measured with a 7-point Likert scale (1 = totally disagree, 7 = totally agree). All Cronbach’s α (see Table 2) were in an acceptable high range of ≥0.70 (Ullman, 2013), except the scale assessing the negative attitude toward robots (α = 0.67).

**TABLE 2:** Means, standard deviations, correlations, and reliabilities of the included variables.

		*M*	*SD*	1	2	3	4	5	6	7	8
1	Propensity to trust	4.97	1.21	(0.70)							
2	Negative attitude	3.40	0.94	–0.12	(0.67)						
3	State anxiety	2.98	0.98	**−0.42**	0.01	(0.76)					
4	Initial learned trust	5.10	0.96	**0.42**	**−0.47**	**−0.59**	(0.85)				
5	Dynamic learned trust t_1_	5.34	0.75	**0.40**	**−0.58**	–0.36	**0.79**	(0.81)			
6	Dynamic learned trust t_2_	5.54	0.84	0.20	**−0.57**	–0.31	**0.72**	**0.85**	(0.87)		
7	Distance t_1_	34.82	24.72	0.10	0.20	0.08	–0.03	0.01	0.26	−	
8	Distance t_2_	18.64	13.79	–0.19	0.30	–0.01	–0.19	–0.04	0.17	**0.58**	−

*Diagonal: Scale reliabilities (Cronbach’s α). Significant correlations in bold (p < 0.05, two-sided test).*

### Statistical Procedure

To test the study hypotheses, scale means were calculated and used for all statistical procedures. The relationships of user dispositions and state anxiety with learned trust and comfort distance were calculated using regression and mediation analysis. Bivariate relationships were tested with the Pearson product- moment correlation. The reported results refer to a one-sided test in the case of directed hypotheses. Changes through repeated interaction were assessed with paired *t*-tests or ANOVAs. All analyses were conducted in R, version 3.6.2. For mediation analysis, the R package *mediation* version 4.5.0 was used as described by Tingley et al. (2014). The mediation effect (indirect effect) was tested with the bootstrapped 95% confidence intervals (CIs) with 5,000 samples (e.g., Hayes, 2018).

#### Preconditions

Regarding preconditions for the applied methods, first, there was no missing data in the overall data set. Second, an outlier analysis along the mean values indicated that the distance measurement of two subjects in the second trial exceeded a z-score of |3.29| (Ullman, 2013; distance = 1.10 m and 1.04 m, Mdn = 0.19 m, IQR = 0.21 m). As mentioned above, these two subjects were excluded from further analysis. All other values did not show any outliers. Third, Shapiro-Wilk tests indicated no significant deviations from a normal distribution for all mean values in the overall sample (all *p* > 0.01). Fourth, the effects of the experimental manipulation of the robot’s appearance (size and manipulator outreach) on the relevant constructs in this study were analyzed using general linear models. The results showed no effects of the experimental manipulations on dynamic learned trust for neither trial (t_1_ and t_2_). On the other hand, the size of the robot had a significant effect on the comfort distance in the first trial, *b* = 27.57, *t*(24) = 2.33, *p* = 0.028, with the distance being larger in the tall robot (*M*_*tall*_ = 0.47 m, *SD*_*tall*_ = 0.18 m) than the short robot condition (*M*_*short*_ = 0.23 m, *SD*_*short*_ = 0.25 m). Thus, the results regarding the hypotheses on relationships with the comfort distance for t_1_ should be interpreted taking the effects of the robots’ size manipulation into account. Furthermore, to rule out biases due to group effects, a series of general linear models was run for each user disposition to check for interactions with the experimental manipulations on the dependent measures. The results showed no significant interactions on dynamic learned trust for neither trial (t_1_ and t_2_).

## Results

Table 2 provides the mean values, standard deviations, reliability, and correlations for all included scales for the complete cleaned sample. Due to the relatively small sample size, only correlations of *r* = 0.40 or higher reached a significant *p*-value (*p* < 0.05) with a two-sided test. According to Cohen (1988), correlation coefficients above *r* = 0.30 are considered as moderate effects. While we do not interpret non-significant results, the effect size might be considered as a preliminary indication of the existence of the respective relationship in the population.

### Interrelation of Trust Layers

**FIGURE 4:**
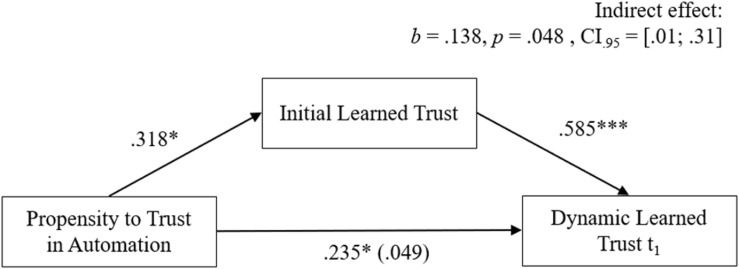
Mediation model for the three investigated trust layers.

The investigated research model proposes positive relationships between the propensity to trust with initial and dynamic learned trust (H1.1). Specifically, a mediation effect from the propensity to trust over initial learned trust on dynamic learned trust was hypothesized (H1.2) to examine the trust formation process and timely sequence of the different trust layers. Accordingly, it was expected that participants would trust the robot more with growing familiarity and experience. Therefore, learned trust in the robot was expected to increase with repeated interaction (H3). Drawn from the correlation coefficients (see Table 2), the propensity to trust was found to be significantly related to initial learned trust (*r* = 0.42, *p* = 0.014) and dynamic learned trust in the first (*r* = 0.40, *p* = 0.018) but not in the second trial (*r* = 0.20, *p* = 0.158). As can be seen in Figure 4, the effect of the propensity to trust on dynamic learned trust in the first trial was fully mediated by initial learned trust, which was substantiated by the significant statistical test of the mediation effect, *b* = 0.138, *p* = 0.048, CI_0_._95_ = [0.01;0.31]. For the development of dynamic learned trust over time, the results of a repeated measure ANOVA supported H3, and indicated a significant linear trend, *F*(1, 27) = 11.50, *p* = 0.002, η^2^ = 0.299, as reflected in increasing means of learned trust at the different points of measurement (regardless of the experimental manipulations): initial learned trust before the interaction (*M_*t*__0_* = 5.10, *SD_*t*__0_* = 0.96), dynamic learned trust in the first (*M_*t*__1_* = 5.34, *SD_*t*__1_* = 0.75) and second trial (*M_*t*__2_* = 5.54, *SD_*t*__2_* = 0.84). These findings support a dynamic increase of learned trust toward a specific robot with emerging familiarity and interaction. Considering the previous assumptions and results, H1.1 and H1.2 can be partly accepted for dynamic learned trust in the first trial and H3 can be fully accepted.

### Effects of User Dispositions and State Anxiety on Initial Learned Trust

**FIGURE 5:**
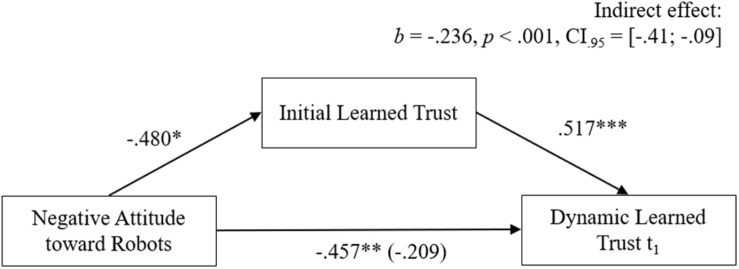
Findings for the mediation from negative attitudes toward robots to dynamic learned trust by initial learned trust.

Hypothesis 2 stated that user attitudes influence learned trust in a robot. Besides the propensity to trust, an effect from negative attitudes toward robots on initial and dynamic learned trust was expected (H2.1). Similar to the mediation effect for the different trust layers, the effect of negative attitudes on dynamic learned trust was hypothesized to be mediated by initial learned trust (H2.2). In favor of H2.1, the correlation coefficients showed significant medium to high negative correlations between negative attitudes toward robots with initial learned trust (*r* = −0.47, *p* = 0.006), dynamic learned trust after the first (*r* = −0.58, *p* < 0.001) and the second trial (*r* = −0.57, *p* < 0.001). In line with H2.2, a mediation analysis showed a significant indirect effect of negative attitudes over initial learned trust on dynamic learned trust in the first trial (*b* = −0.236, *p* < 0.001, CI_0_._95_ = [−0.41; −0.09]; see Figure 5).

Above this, the results of the regression analysis with both dispositional predictors revealed a significant influence of both propensity to trust, β = 0.350, *t*(25) = 2.24, *p* = 0.034, and negative attitude toward robots, β = −0.408, *t*(25) = −2.61, *p* = 0.015, on initial learned trust. The model accounted for 29.78% of variance in the criterion.

Regarding initial learned trust, it was furthermore assumed that people who feel more anxious in anticipation of an imminent interaction with a robot would have less trust in the robot before initially interacting with it (H4.1). Furthermore, this research’s overall theoretical assumption was that affect before a direct interaction is related to the initial trust level. In turn, the latter is proposed to constitute a basis for dynamic learned trust in the early interaction with a robot. This influence of initial learned trust on dynamic learned trust is assumed to be replaced by more performance-related new information in subsequent ongoing interaction with a robot. Therefore, a decreasing correlation strength was expected from initial learned to dynamic learned trust and from the first to the second trial (H4.2). In accordance with H4.1 and H4.2, the correlation coefficients between state anxiety and learned trust showed a decrease over time, with a highly significant negative correlation between state anxiety and initial learned trust (*r* = −0.59, *p* < 0.001), and a medium significant negative correlation with dynamic learned trust in the first trial (*r* = −0.36, *p* = 0.031), and a non-significant negative correlation in the second trial (*r* = −0.31, *p* = 0.054). The results thus supported H4.

**FIGURE 6:**
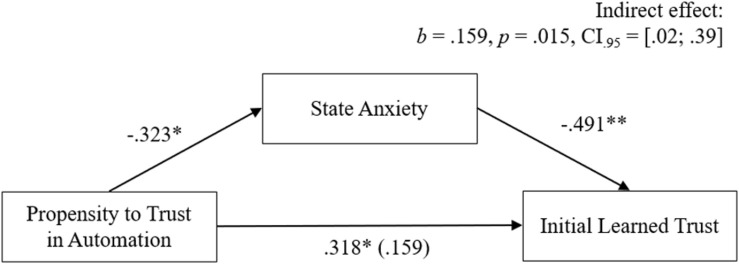
Findings for the mediation from the propensity to trust to initial learned trust by state anxiety.

As additionally assumed in H5.1 and H5.2, the effect of the two dispositions on initial learned trust was expected to be mediated by the current affective state in the situation. To test these effects, parallel-mediation analyses were computed with the respective disposition as predictor, initial learned trust as criterion and state anxiety as mediator. In line with H5.1, the statistical test of the mediation effects (see Figure 6) showed a full mediation from propensity to trust on initial learned trust through state anxiety, indicated by a significant indirect effect, *b* = 0.159, *p* = 0.020, CI_0_._95_ = [0.02;0.39]. Due to the non-significant relationship between negative attitude toward robots as predictor and state anxiety as mediator (*r* = 0.01, *p* = 0.472), which is a prerequisite for calculating mediation analysis, no mediation for this effect was tested. Thus, in this research H5.2 could not be supported.

### Effects of Learned Trust on Distancing Behavior

Hypothesis 6 proposed that people with lower trust in the robot prefer to keep more distance when being approached by the robot. Dynamic learned trust and the comfort distance were measured repeatedly at t_1_ and t_2_. As shown in the correlation matrix (Table 2), the comfort distance at neither t_1_ nor t_2_ was significantly related to any of the remaining variables. Therefore, H6 has to be rejected.

Besides, in accordance with the findings of trust increase over time, it was assumed that participants would let the robot approach closer with growing familiarity and experience. Therefore, the comfort distance toward the robot was expected to decrease from the first to the second trial (H7). Participants in fact allowed the robot to come closer with repeated interaction (*M*_t__1_ = 0.35 m, *SD*_t__1_ = 0.25 m; *M*_t__2_ = 0.19 m, *SD*_t__2_ = 0.14 m). The results of a paired *t*-test showed a significant result, *t*(27) = 4.24, *p* < 0.001, *Cohen’s d* = 0.808. Therefore, an overall main effect was supported by the reported findings. H7 can thus be accepted.

## Discussion

This research investigated the early trust development toward an unfamiliar service robot in a domestic environment prior to and during initial familiarization. Especially, the interrelation of different layers of trust was investigated, as well as the foundation of differences in learned trust in the robot in both user dispositions and affective user states. Furthermore, the role of these variables for interindividual differences in proximity preferences was investigated.

### Summary of Results

Taken together, the results of the study supported eight of the eleven study (sub-)hypotheses. First of all, in line with the investigated research model and the assumption of different trust layers, the dispositional propensity to trust was positively related to the two layers of initial and dynamic learned trust (H1.1). Furthermore, the relationship between propensity to trust and dynamic learned trust was mediated by initial learned trust in the robot in the first trial (H1.2). Besides the propensity to trust, negative attitudes toward robots were negatively related to both initial and dynamic learned trust (H2.1). Similarly, the relationship between negative attitudes and dynamic learned trust was mediated by initial learned trust in the robot (H2.2). The results emphasize that domain-specific user dispositions, to some extent, influence trust ratings in the early interaction with unfamiliar technologies. The importance of this embeddedness of specific trust in user dispositions is especially emphasized by the large proportions of variance in initial learned trust, explained by the propensity to trust in automation and negative robot attitudes. Above this, in accordance with H3, learned trust in the robot increased throughout the experiment. The finding underlines that a familiarization effect takes place relatively quickly, and that lay users can get used to domestic robots after a short period already. This was further supported by a decreasing distance participants kept with repeated trials (H7).

Besides the formation of trust with emerging familiarity, the findings underline the notion that learned trust in a robot is affected by the experience of anxiety prior to the interaction with a robot. Thereby, a declining strength of the relationship between state anxiety and trust over time was found. While initial learned trust was strongly affected by the initial level of anxiety (H4.1), dynamic learned trust after the two trials showed diminished relationships with state anxiety (H4.2). Most interestingly, the relationship between the general propensity to trust in automated technology and initial learned trust in the robot was mediated by the user’s initial level of state anxiety (H5.1). In contrast, no similar mediation effect for negative attitude toward robots was found (H5.2). Finally, the comfort distance toward the robot was not correlated with any of the investigated psychological constructs, contradicting H6.

Overall, the findings highlight the role of personality differences and individual variances in the technology-related learning history for the experience of anxiety and the formation of trust in automation. On this basis, a consideration of these findings in robot development and design can favor positive interaction outcomes such as safe usage, appropriate trust, and comfortable interaction. Before illustrating practical implications, this research’s theoretical contributions are discussed in more detail.

### Interrelation of Different Trust Layers

The reported findings underline the relevance of different layers of trust in automation, as proposed, for example, by Hoff and Bashir (2015). The findings demonstrated that inter- and intraindividual trust variations originate from both individual trait differences in the tendency to trust automation (propensity to trust in automation) and in the trust-related learning process prior to and during the interaction with a robot (initial and dynamic learned trust). In line with the propositions of the Three Stages of Trust framework (Kraus, 2020), this interplay of different trust layers in adopting a formerly unfamiliar robot is supported by the mediation cascade from the propensity to trust in automation via initial learned to dynamic learned trust. In accordance with described relationships between dispositional and system-specific trust in other domains (e.g., Lee and Turban, 2001; Merritt and Ilgen, 2008; Merritt et al., 2013; Kraus et al., 2020a), this mediation supports a timely order of these three trust layers throughout the familiarization process with an automated system like the investigated robot. The propensity to trust as a technology-specific personality trait reflects the sum of learning experiences with automated technology and considerably determines the expectations with which an individual enters the familiarization with a newly introduced system. Based on this personality variable, the available information prior to the first interaction with the system under consideration is used to build up a level of initial trust, which in turn builds the starting point for the trust calibration process during the actual interaction. Taken together, this research supports the notion of user dispositions and different trust layers that build onto each other in the emergence of a specific level of dynamic learned trust at a given time during the interaction with a robot.

Above this, in line with earlier research in HRI (e.g., van Maris et al., 2017; Yu et al., 2017) and the interaction with other automated technology, for example, plant simulations (Lee and Moray, 1994) and automated driving (e.g., Beggiato et al., 2015; Hergeth et al., 2015; Kraus et al., 2019b), in this study, trust in the robot was found to increase throughout the interaction incrementally. As long as there is no negative information like an experience of restricted reliability (e.g., automation malfunction; Kraus et al., 2019b), a violation of initial expectations, or realization of initial concerns and fears, accumulated positive information and experiences lead to an increase in trust over time. At the same time, this shows that despite the discussed differences between systems from different technological domains, general results from other domains might be transferable to HRI.

Derived from that, researchers must consider carefully which trust layer and which points in time are addressed in their experimental design. Notwithstanding, trust should be measured several times throughout HRI research. Furthermore, a combined consideration of dispositional trust and learned trust should be entailed in research designs. Taken together, the investigation of factors affecting the process of trust formation and calibration, in which these three trust layers build on each other, is an essential prerequisite for predicting, understanding, and modifying the interaction with a robot at a given point in time.

### Role of User Dispositions and States for Trust in Human-Robot Interaction

This research identified two user dispositions and the emotional state anxiety to affect trust processes, answering the call for a more thorough investigation of user characteristics’ influence in HRI (e.g., Hancock et al., 2011). Thereby, the findings go beyond previous research and emphasizes the meaningfulness of (technology-)specific personality traits and attitudes in the individual reaction to technology.

The presented study supports a relationship between state anxiety and learned trust in service robots in line with the affect-as-information approach. Robots are a new technology and most people (our sample in particular) did not have many opportunities to establish first-hand experiences. Therefore, it is not surprising that anxiety plays an essential role in the initial familiarization process with robots in the face of the associated uncertainty (and maybe also pre-existing reservations). Besides the actual anxiety, which is directly triggered by the new, unpredictable robot, also misattributions of affective states might influence trust and other evaluative outcomes. The study findings corroborate the results of studies on human interactions, in which mood-congruent judgments of trust in a co-worker (Dunn and Schweitzer, 2005) or general life satisfaction (Schwarz and Clore, 1983) were found. Interestingly, in Schwarz and Clore’s (1983) work, participants only (mis)attributed their bad mood onto judgments about their lives, when no alternative (external) transient source for attributing the bad mood to was salient. In light of the presented findings, the robot offers a plausible source for the attribution of current feelings (and is at the same time one cause for these; see also in section “Reflections on Trust Formation and Calibration Processes at Different Points in Time”). In line with other research (Stokes et al., 2010; Merritt, 2011), the results emphasize the need to consider affective states and mechanisms of information processing, especially in early interactions with new technology when the user has not yet had any experiences of his own with the system to build trust on. With growing familiarity, users start to build more on personal experiences than potentially misattributed and misinterpreted inner states.

Furthermore, state anxiety was found to be predicted by the propensity to trust in automation. Also a significant trait-state mediation from the propensity to trust to initial learned trust via state anxiety was found. Overall, the reported findings on anxiety point into the direction that individual differences in the general tendency to trust shape the affective reaction to new technology which in turn influences initial trust levels. To conclude, for understanding the interindividual variances in the reaction toward a specific robot, a consideration of pre-existing individual differences in the propensity to trust in automation and the consideration of the individual learning history and affective reaction seems worthwhile.

### Findings on Distancing Behavior

In light of the affect-regulating function, this research assumed the user’s trust level to serve as an information and evaluation source for the comfort distance toward the robot. However, no relationship between the behavioral measurement and trust in the robot could be found. At the same time, findings indicate a decrease of the comfort distance toward the robot over time.

There are different potential explanations for these findings. First, it should be considered that the study sample was relatively small since real face to face interactions and behavior were investigated. As a result, correlations in the area of moderate effect sizes did not reach significance. Furthermore, it seems plausible that the small to medium correlations between trust and distancing behavior are mediated and moderated by interposed processes and constructs, which were not addressed in this study. Besides, this study applied a robot-human approach with participants instructed to indicate their preferred distance. A different design (e.g., human-robot approach, field observation) might have produced other results because users could adjust the actual distance more dynamically and adapt it to the interaction context, task, and shifting inner states.

Second, there might be a direct effect of robot characteristics on proximity preferences, which is not mediated by trust. A multitude of research supports that robot-related factors influence the preferred distance toward a robot. A potential direct effect of robot characteristics on distance preferences was supported in this study (see Miller et al., 2021). Besides this, the findings underline an important role of the subjective perception of robot characteristics on the initial evaluation of and reaction to robots. Specifically, this is emphasized by the correlations of both state anxiety and the investigated trust layers with different robot evaluations (especially uncanniness).

### Reflections on Trust Formation and Calibration Processes at Different Points in Time

Regarding the process of trust formation and calibration over time within different phases of familiarizing with robots and other new technology, some of the presented findings are worth to be discussed in more detail. A closer inspection of the magnitude of bivariate correlations between dispositions, state anxiety, and learned trust at different times of measurement (Table 2) reveals an interesting pattern. On the one hand, the strengths of both the relationships of the propensity to trust and state anxiety with learned trust decreased over time. On the other hand, the correlations between (negative) attitudes and learned trust increased in their magnitude over time. Besides, the negative attitude toward robots was not related to state anxiety, implying no similar mediation effect on learned trust as for the propensity to trust. These findings point toward differential information sources and information processing mechanisms, through which trust in a specific system is built and calibrated. In the following, two possible explanations for changing information use at different times in the familiarization with automated systems are discussed, which can account for the observed patterns: changing availability of information (sources) and different processing mechanisms depending on motivation and cognitive capacities.

First, it is reasonable to assume that different kinds of information are present at different phases during the familiarization with a new technological system. Before system use, mainly information from second-hand testimonials of users, marketing, and information campaigns are available. In contrast, when interacting with a system, the actual system behavior and user interface output provide diagnostic information to assess a system’s trustworthiness. Accordingly, in the early phase of getting to know an automated system (before actual system use), the available information tends to be more vague, indirect, and unspecific. On the contrary, in the later phase(s), the information results from a direct first-hand experience of the user and tends to be associated with system behavior, the current task, and environmental conditions. Therefore, it seems reasonable to assume that the relevance of different categories of trust cues (reputation-, purpose-, process-, performance-, and appearance-related; see Kraus, 2020) changes over time. At this point, the differential impact and character of available trust cues prior and during the interaction with robots have to our knowledge not been extensively investigated and, therefore, provide promising directions for future research.

The second plausible mechanism of differential information use prior and during the interaction is a result of the characteristics of information processing in attitude formation and change. This is related to the idea of different routes of information processing by Lee and See (2004; affective, analogous, analytical) and the assumptions of dual-process theories of attitude formation and change (Chen et al., 1996; Chaiken and Trope, 1999). Dual-process theories assume two routes through which information can be processed and through which change of attitudes is initiated. For example, the Elaboration-Likelihood Model (ELM; Petty and Cacioppo, 1986) proposes a central route, through which an effortful analysis of the meaning of information is conducted (bottom-up). On the contrary, the peripheral route represents an attributional process, in which surface characteristics of the information or the information source lead to the change of attitudes (top-down). An essential prediction of the ELM (and other dual-process theories) is that the motivation and the ability of the person in focus determines which route of information processing is used to which extent (Petty and Cacioppo, 1986). The relative contribution of the two processes in trust formation and calibration is expected to change according to the information available and the associated affective, cognitive, and motivational processes at play at different points in time. In support of this, Kraus et al. (2019a) found that participants used information provided before the first interaction with an automated driving system differently in building up their trust levels based on their individual expression of *need for cognition*, which reflects an individual tendency to enjoy and engage in cognitive tasks (Cacioppo et al., 1984) and thus for effortful analysis of provided information (e.g., Cacioppo et al., 1983). From this, it can be derived that, irrespective of the availability of information, other characteristics of information and, therefore, different entailed trust cues might be used in different trust formation phases. Based on these changes in information processing, it can further be assumed that the extent to which the user’s self-monitoring of, for example, psychological states (e.g., workload, stress, affect) is used as a source for trust changes over individuals and time in the trust formation process. For example, if users are not motivated or have restricted capacity to reflect (which might be the case in trust processes prior to an interaction), they might more strongly build their trust on their current feeling (e.g., affective state, “How do I feel about it?”) in the sense of affect-as-information (e.g., Schwarz and Clore, 1988). Similarly, in such a situation, users might tend to engage more strongly in top-down than in bottom-up processing and exemplarily base their trust formation more on already existing general attitudes (e.g., attitude toward robots in general). On the contrary, in calibrating one’s trust during (repeated) system use, the motivation to correctly assess provided trust cues to use the system adequately should be drastically increased due to the associated higher risks. As a result, the influences of affect and prior attitudes on trust might be diminished in favor of actual diagnostic trustworthiness (bottom-up) information available in a situation.

The results of this study suggest such interaction of different routes of information processing and a differential role of top-down and bottom-up processes in trust formation and calibration. Findings indicate that the affective and cognitive trust processes change in their relative importance for explaining the momentary level of learned trust. While at the beginning of the interaction, trust is more strongly affected by state anxiety and the propensity to trust (which reflect top-down processes), the influence of prior negative attitudes toward robots remains in the same range. While the proportions of variance explained by anxiety reflect the affective component of trust, the negative attitudes toward robots might reflect a more cognitive evaluative trust basis. If these attitudes are of rational nature, they might also gain relevance in effortful cognitive decision-making. Taken together, the interactive role of different information processing over time in trust formation is a promising direction for further research that might essentially contribute to an understanding of trust processes and to an appropriate design of information about robots, robot appearance, and behavior, as well as HRI in practice.

### Practical Implications

Since trust in robots is essential to foster safe, efficient, and comfortable interactions, the reported results provide a foundation for the derivation of design recommendations in developing service robots for domestic environments in particular.

First of all, as reflected in personality traits and technology-related attitudes, user dispositions were found to affect both the experience of anxiety in the face of an initially unfamiliar robot and the individual trust level. This underlines the importance of taking the target group and individual user into account when designing interactions with robots. Engineers and designers might carefully consider which users they develop the robot for and if a possible differentiation of user groups in terms of their personality and attitudes is reasonable. In practical settings, users with initial negative attitudes might be addressed by providing more information on the robot’s potential advantages. In contrast, it is more important for users with a positive attitude to ensure that they do not overtrust the robot and overestimate its abilities by providing transparent and adequate information about the robot’s limitations. Furthermore, the investigated user dispositions and the level of anxiety might be assessed to individualize the process of introducing a robot and personalizing the robot’s interaction concept. In this way, first interactions with robots, for example, for novices vs. tech-savvy users, could be set up differently and adapted to individual needs. While for novice users, anxiety-reducing interaction strategies and behaviors might be appropriate, as for example, the safe planner introduced by Kulić and Croft (2005), experienced users might already be accustomed and habituated to the movements and operating modes of the robot and therefore accept closer proxemics right away. Consequently, a customized robot might be less scary, more acceptable, and efficient, and might facilitate trust calibration.

Moreover, the reported findings emphasize the importance of how robots are advertised and promoted before handed over and entering personal spaces. To facilitate a certain level of trust in the robot, demonstrating its capabilities, functioning, and potential benefits and limitations and risks should occur beforehand to minimize anxious feelings before the first interaction with the new companion. The goal should be conveying a realistic picture of potential threats so that the users can rely on facts rather than on possibly misattributed feelings of arousal and anxiety. Taken together, while consideration of characteristics and appearance of the robot in design is essential, the individual reaction to and evaluation of a robot is considerably influenced by pre-existing differences regarding user personality and the individual learning history with technology and robots. A more detailed discussion of how personality differences can be considered in user education and the design of automated systems can be found in Kraus et al. (2020a) and Kraus (2020).

Additionally, in line with the Three Stages of Trust framework by Kraus (2020), this research underlines that different trust layers are involved in the emergence of learned trust in a robot at a specific point in time. This includes the propensity to trust in automation, which constitutes a specific personality trait and is, besides others, influenced by the individual learning history with technology. Therefore, in practice, it seems relevant to understand the users’ level of expertise and design the introduction process, the provided information, and the user interface accordingly. As mentioned, the introduction might be shorter and more about the technical functioning for experienced users or technological experts. At the same time, the interface might provide different information or enable other functions with growing experience.

Furthermore, while the experiences during the actual interaction with a robot are undoubtedly important for the individual level of dynamic learned trust, the level of trust established prior to the interaction determines user expectations during the interaction with a robot. Similarly, the information provided before any interaction influences how users interpret the robot’s behavior during HRI. Hence, trust processes and the available information before the actual interaction (initial learned trust) with a robot need to be considered to understand how trust in a robot at a certain point during the interaction (dynamic learned trust) is established. Therefore, both researchers and designers might consider the following questions in the understanding of the formation of learned trust in a specific automated system under consideration. What image is (currently) conveyed by media reports, how is the specific robot advertised, what information is available online and what are experiences reported by family or friends? All these information sources shape a robot’s evaluation and the trust level before the user and robot even met or interacted, influencing the perception, evaluation, and reaction in HRI.

Furthermore, this study’s findings underline the relevance of different types of information and different psychological processes for trust at different phases of the trust formation and calibration process. While more research is necessary to gain further insights into the relative importance of different kinds of information and information processing mechanisms at these different phases, at this point, it seems essential to focus on personality and user state effects in the initial phase of trust formation. Therefore, for user education and design of HRI, individual anxiety might be addressed more at the beginning of the interaction. In contrast, general attitude-based trust formation seems relevant for trust formation throughout all phases of early interaction. Therefore it is vital that positive attitudes are promoted by emphasizing a robot’s assistance potential. This could be achieved by providing transparent information about the robot’s capabilities and limitations or repeated HRI with positive experiences (building a personal learning history).

Additionally, the results show that users allow a robot to come comparatively close and act in immediate proximity to the user. Since domestic robots are likely to work and collaborate in close physical proximities with users as compared to industrial robots, this finding is of high practical relevance. It allows developing and employing robots for tasks that require close collaboration between robots and humans (like, e.g., in healthcare applications). Technical restriction (of, e.g., recognition systems) and resulting perceived impairment of robot performance should be considered here (e.g., Mead and Matarić, 2015). Furthermore, individual user characteristics and preferences could also be considered in robots’ proxemic design, for example, by robots recognizing whether they have already interacted with a user or not (e.g., van Oosterhout and Visser, 2008). In this regard, reactions from preceding interactions could similarly be used to adapt the robot behavior dynamically.

### Strengths, Limitations, and Future Directions

On the side of this research’s strengths, specifically, the real-life interaction with a (domestic) service robot in the experimental setup has to be stressed. A second advantage of the study design is the combination of subjective variables and objective behavioral outcomes. A further strength is the derivation of the study hypotheses from theorizing in trust in automation, HRI, and broader psychological domains. This facilitates the scientific accumulation of knowledge about the interaction and the design of service robots. Furthermore, the results guide practitioners with various implications for the interaction design between humans and robots regarding the promotion of calibrated trust and adequate proxemic behavior.

Nevertheless, like all research, this study has some limitations, which might be addressed in future research. First, the investigated effects of familiarization are likely to not fully play out during the observed time frame over two directly sequential interactions. A longitudinal design with actual interaction and task completion might be implemented in future studies to show the effects under consideration in the long term, like for example in Koay et al. (2007). Second, changes in state anxiety were not assessed in the experiment. In a similar manner, the consideration of different user states, such as arousal or positive and negative affect, could have strengthened the findings. In this context, the application of physiological methods is a promising approach, for example, to draw conclusions on anxiety or stress (e.g., Crossman et al., 2018). Third, as the sample was self-selected, particularly anxious people might be underrepresented in the study. Future studies could take further steps to include anxious participants. Fourth, only one personality trait and one dispositional technology attitude were included in this study. Future research could address the relationships of further dispositional variables with trust and distance preferences. For example, the role of attitudes toward technology in general and robots in particular might be considered as well as (robot) trait anxiety. Fifth, between the two trials an experimental manipulation of the robots’ size and manipulator position took place, which might additionally produce interindividual variance and changes in trust and distance (see results in Miller et al., 2021). Sixth, the sample size of this study was rather small for a correlative design as reflected in restricted power. While this was an effect of the natural experimental setting of the study, the reported significant findings underline the large effect sizes of the relationships between the variables entailed in the investigated research model.

Apart from that, this research provides an integrative theoretical basis for further consideration of the role of psychological variables associated with interindividual variances in trust in robots and HRI. On this basis, further studies aiming at a more detailed understanding of the psychological process in which trust in robots is formed and calibrated might focus on additional dispositional, situational, and robot-related variables, as well as the psychological processes, in which these interact over time. This involves interactions between dispositional user characteristics and situational variables (e.g., the role of robot expertise in interpreting a robot’s distancing behavior) and interactions between dispositional variables and robot design features (like the role of cooperative personality traits for preferences of different levels of robot anthropomorphism). Additionally, future research might strive to validate the established relationships in a large-scale sample. Also, the transferability of the findings to other than domestic environments could be addressed in further studies. Finally, the results of this study raise the question if users get used to robots very quickly and therefore the implications for overconfidence might be addressed in future research. Future studies may focus on an appropriate and calibrated level of trust in robots and how this can be achieved. It remains to be seen in the next years how and on what basis social norms with robots (that have been established in the interaction between humans over decades) will develop when these are increasingly integrated into personal spaces and society.

## Conclusion

Service robots are increasingly entering public and private spaces, which will promote close and personal interactions. This research strived to further investigate the role of user characteristics in the emergence of trust and distancing behavior in HRI. Especially the a priori attitude toward robots in general and the propensity to trust in automation seem to contribute to the understanding of interindividual differences in trust in robots and therefore affect appropriate robot use. By integrating psychological antecedents of close human-robot collaborations such as personality traits, affect and trust, this research provides a foundation for designing robots and directions for future developments. A role of a mediation mechanism from user dispositions to learned trust by state anxiety was supported. Thus, this research contributes to a deeper understanding of underlying determinants for affective and behavioral reactions in close personal interaction with robots. Taken together, the reported findings support central propositions of the Three Stages of Trust framework (Kraus, 2020) in terms of the history-based psychological process in which trust in automated systems is built up and calibrated. In this regard, the reported findings argue for considering user dispositions and processes before the actual interaction with a specific robot to understand better how evaluations and decision-making regarding one particular robot are established on a psychological level. The presented research constitutes a starting point for further research on the psychological basis of trust in robots by integrating broader personality traits, robot-related traits, and trust constructs with different specification levels and foci simultaneously. This research provides a foundation for utilizing the benefits and potentials of robots more fully and successfully integrating robots into our society and everyday life.

## Data Availability Statement

The datasets generated for this study are available on request to the corresponding authors.

## Ethics Statement

This study was conducted with the consent of the ethical committee of Ulm University (approval no. 95/19). The ethical approval was granted under the condition that the data protection regulations were adhered to. The participants provided their written informed consent to participate in this study.

## Author Contributions

LM collected the data, performed the data analysis, and led the manuscript write-up. JK generated the research questions and study method, led the study implementation, and had a substantial part in writing and editing the manuscript. FB was substantial to study conception, design, and implementation and assisted with the manuscript write-up. MB assisted with the manuscript write-up. All authors were involved in the research process.

## Conflict of Interest

The authors declare that the research was conducted in the absence of any commercial or financial relationships that could be construed as a potential conflict of interest.
